# Evolutionary Mirages: Selection on Binding Site Composition Creates the Illusion of Conserved Grammars in *Drosophila* Enhancers

**DOI:** 10.1371/journal.pgen.1000829

**Published:** 2010-01-22

**Authors:** Richard W. Lusk, Michael B. Eisen

**Affiliations:** 1 Department of Molecular and Cell Biology, University of California Berkeley, Berkeley, California, United States of America; 2 Genomics Division, Ernest Orlando Lawrence Berkeley National Laboratory, Berkeley, California, United States of America; 3 California Institute of Quantitative Biosciences, University of California Berkeley, Berkeley, California, United States of America; 4 Howard Hughes Medical Institute, University of California Berkeley, Berkeley, California, United States of America; Stanford University School of Medicine, United States of America

## Abstract

The clustering of transcription factor binding sites in developmental enhancers and the apparent preferential conservation of clustered sites have been widely interpreted as proof that spatially constrained physical interactions between transcription factors are required for regulatory function. However, we show here that selection on the composition of enhancers alone, and not their internal structure, leads to the accumulation of clustered sites with evolutionary dynamics that suggest they are preferentially conserved. We simulated the evolution of idealized enhancers from *Drosophila melanogaster* constrained to contain only a minimum number of binding sites for one or more factors. Under this constraint, mutations that destroy an existing binding site are tolerated only if a compensating site has emerged elsewhere in the enhancer. Overlapping sites, such as those frequently observed for the activator Bicoid and repressor Krüppel, had significantly longer evolutionary half-lives than isolated sites for the same factors. This leads to a substantially higher density of overlapping sites than expected by chance and the appearance that such sites are preferentially conserved. Because *D. melanogaster* (like many other species) has a bias for deletions over insertions, sites tended to become closer together over time, leading to an overall clustering of sites in the absence of any selection for clustered sites. Since this effect is strongest for the oldest sites, clustered sites also incorrectly appear to be preferentially conserved. Following speciation, sites tend to be closer together in all descendent species than in their common ancestors, violating the common assumption that shared features of species' genomes reflect their ancestral state. Finally, we show that selection on binding site composition alone recapitulates the observed number of overlapping and closely neighboring sites in real *D. melanogaster* enhancers. Thus, this study calls into question the common practice of inferring “*cis*-regulatory grammars” from the organization and evolutionary dynamics of developmental enhancers.

## Author Summary

Because mutation is a random process, most biologists assume that apparently non-random features of genome sequences must be the result of natural selection acting to create and preserve them. Where this is true, genome sequences provide a powerful means to infer aspects of molecular, cellular, and organismal biology from the signatures of selection they have left behind. However, recent analyses have shown that many aspects of genome structure and organization that have traditionally been attributed to selection can often arise from random processes. Several groups—including ours—studying the sequences that specify when and where genes should be produced have identified common, seemingly conserved, architectural features, based on which we have proposed new models for the activity of the complex molecular machines that regulate gene expression. However, in the work described here we simulate the evolution of these regulatory sequences and show that many of the features that we and others have identified can arise as a byproduct of random mutational processes and selection for other properties. This calls into question many conclusions of comparative genome analysis, and more generally highlights what Michael Lynch has called the “frailty of adaptive hypotheses” for the origins of complex genomic structures.

## Introduction

The transcriptional output of developmental enhancers is affected by the spatial organization of the transcription factor binding sites they contain. The relative positioning of sites is known from individual cases to modulate direct competition between factors for the same site [1],[2], cooperative and repressive interactions between transcription factors [3],[4], and the formation of higher-order regulatory complexes [5]–[7]. However, we have a precise understanding of the relationship between binding site organization and function for few, if any, developmental enhancers.

In the absence of efficient experimental protocols for dissecting enhancer function, recent efforts have attempted to infer functional constraints on binding site organization from the distribution and evolution of binding sites in enhancers of interest. We recently examined developmental enhancers in species distantly related to *D. melanogaster* and found a strong preferential conservation of overlapping and proximal sites [8], a result which was confirmed by a recent survey of enhancer evolution across the twelve sequenced *Drosophila* genomes [9]. Others have focused on the density of overlapping and proximal sites, finding that both are significantly enriched [10],[11]. All of these studies, including ours, reached a similar conclusion: the evolutionary dynamics of binding sites in developmental enhancers suggest that clustered and/or overlapping sites are common functional necessities for enhancer activity.

This shared conclusion was premised on the idea that the observed non-random arrangement of sites must be a result of selection on the relative positioning of sites within enhancers. However, alternative explanations for these phenomena, especially the possibility that such arrangements might arise as a byproduct of other mutational and selective pressures [12], have not been explored.

Here we simulate the evolution of real and synthetic *D. melanogaster* enhancers constrained only to maintain their binding site composition and investigate the spatial organization of binding sites within enhancers evolving with no direct selection on the arrangement of sites within them. We show that this simple global constraint on enhancer composition is sufficient to produce many of the organizational and evolutionary features observed in real enhancers, including enrichment and apparent conservation of overlapping and clustered sites.

## Results

### Simulating enhancer evolution

**Figure 1:**
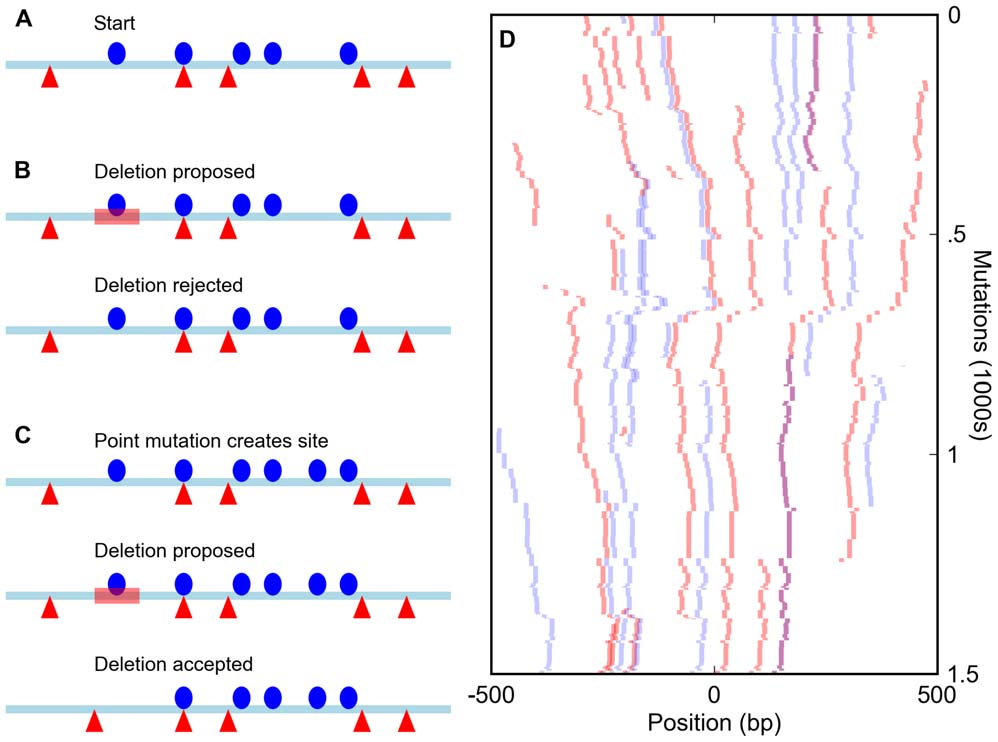
Simulation of enhancers under a compositional constraint. (A) Starting state for a simulation of an enhancer constrained to have five sites for each of two different transcription factors (red triangles and blue circles). (B) A deletion (red bar) eliminates a site, bringing the total number for that factor to four and leading to the rejection of the mutation. (C) A mutation creates a new site (bringing the total to six) and is accepted. The subsequent deletion of an original site (red bar) does not reduce the total below five and is accepted, leading to a binding site turnover event. (D) Sample run of a simulation of an enhancer required to have five sites each for the *D. melanogaster* transcription factors Bicoid and Krüppel over 1,500 mutation-selection rounds. The course of the simulation proceeds from top to bottom, with all Bicoid sites in the enhancer shown in red and Krüppel sites in blue. Overlapped BCD/KR sites are darker and purple.

We used simulations to explore the properties of enhancers evolving under selection on binding site composition. We subjected synthetic enhancers, in which a predefined number of binding sites for one or more transcription factors were randomly positioned in randomly generated sequence with the same composition as *D. melanogaster* non-coding DNA, to mutations sampled from the distribution of substitutions, insertions and deletions observed in *D. melanogaster* [13]. We applied a strong selective constraint to these mutated sequences. If the number of sites in the enhancer fell below a specified threshold, we rejected the new sequence. Otherwise, it was carried through to the next mutational step (Figure 1).

Because such a strict cutoff might not be realistic, we compared the results of these simulations to those involving a large population of enhancers in which suboptimal sequences were assigned a fitness penalty rather than immediately removed. None of the measures of binding site distribution and evolution discussed below differed appreciably between these models (see Text S1). Since these population simulations required significantly greater computational resources, we present only the results of the simpler model below.

### Binding site turnover

The most basic property of our model of enhancer evolution is that most mutations that destroy a binding site will be rejected, as they bring the number of sites present in the enhancer below the specified fitness threshold (Figure 1B). However, the small size of most binding sites means that they are generated *de novo* by random mutation at an appreciable rate. And, once new sites are generated, mutations that destroy existing sites will be tolerated (Figure 1C), leading to non-homologous site conservation, or “binding site turnover” [14]–[17].

**Figure 2:**
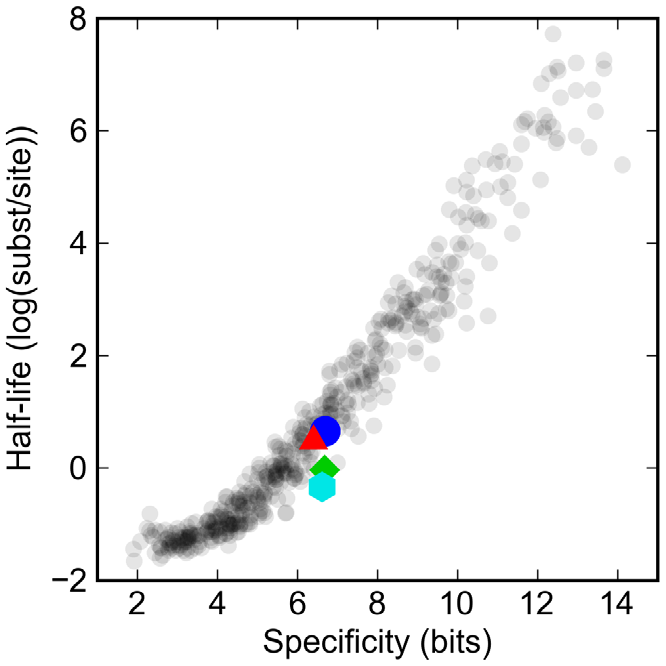
Turnover rates vary widely in proportion to information content of transcription factor binding site model. The log of the half-life of different artificial and real binding sites against their specificity. Synthetic binding sites are plotted in gray, while sites derived from *Drosophila* transcription factors are highlighted: Krüppel (blue circle), Bicoid (red triangle), Giant (green diamond), and Hunchback (cyan hexagon). Specificity is defined as the difference in the information between the binding site and a random sequence of the same length.

The rate at which mutations destroy existing sites for a given factor and create new ones depends on the size and degeneracy of the site recognized by the factor. To examine how specificity affects these rates, we simulated the evolution of enhancers constrained only to have a single site matching real, or randomly generated, transcription factor specificities. The rate of turnover varied considerably, depending on the size of the recognition site, its base composition and degeneracy (Figure 2), with the variance primarily explained by the rate at which new binding sites are generated from random DNA. Longer, less degenerate and GC-rich sites are generated from random sequence at a lower rate and thus turn over more slowly.

The expected half-life (measured in mutational distance) of binding sites for the typical *D. melanogaster* transcription factor was between one and two substitutions per site, or around 50 to 100 million years. This is consistent with previous studies of turnover rates for functional sites in real enhancers that have estimated that there have been around one to two turnover events per site per hundred million years [17],[18].

### Selection on binding site composition alone leads to conserved structure in enhancers

Some transcription factors, such as the *D. melanogaster* proteins Bicoid (BCD) and Krüppel (KR), overlap in their binding specificities, so that the same bases can be part of binding sites for multiple factors [19]–[21]. In specific cases competition between BCD and KR for overlapping sites plays an important role in producing specific expression patterns [22],[23]. The high frequency of overlapping BCD and KR sites in other embryonic enhancers has been used as evidence for the generality of this mechanism [10].

**Figure 3:**
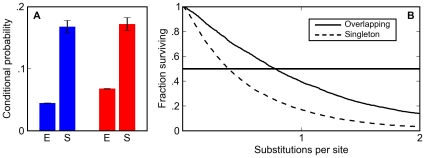
Overlapping binding sites are enriched and appear preferentially conserved in simulated sequences. (A) The post-simulation (S) probability of observing a Krüppel site conditioned on seeing a Bicoid site (blue) and a Bicoid site conditioned on seeing a Krüppel site (red) is always significantly higher than the expected probability (E) in random DNA for binding matrices derived from *in vitro* footprinting experiments. (B) Overlapping sites (solid line) are more likely than isolated sites (dashed line) to persist in simulations at a wide range of mutational distances.

However, when we simulated the evolution of synthetic 1,000 bp enhancers constrained to contain five BCD and five KR sites, we find an almost twofold elevation in the frequency of overlapping BCD and KR sites compared to the random expectation (Figure 3A). Thus selection acting to preserve enhancer composition alone indirectly leads to “higher order” structure in enhancers. This phenomenon is not specific to BCD and KR: rather it is a general property of factors with overlapping binding specificities (data not shown).

The increase in the density of overlapping sites is almost entirely due to their increased half-life relative to isolated sites. In the BCD/KR simulations described above, which had no explicit selection to maintain overlapping sites, overlapping sites persisted 1.5 to 2.0 times longer (depending on the specific choice of matrix) than isolated sites (Figure 3B, Figure S1, and Figure S2).

This difference in half-life between overlapping and isolated sites not only increases the density of overlapping sites; it also significantly alters how they are classified in comparative genomic analyses. Their longer half-life means that overlapping sites are more likely to be found at orthologous positions in related species. In particular, at evolutionary distances in the range typically used for comparative analyses (around one substitution per site) the likelihood of finding an orthologous overlapping pair of BCD and KR sites is two times larger than the likelihood of finding an orthologous singleton site (Figure 3B, Figure S1, and Figure S2). Thus, our simulations show that selection to maintain enhancer composition not only leads to an increase in the density of overlapping sites, it also makes it appear that selection is acting to specifically preserve them.

### A deletion bias induces conserved binding site clustering

Binding sites in real enhancers are clustered, with an excess of short inter-binding-site distances at the expense of long ones [10],[11]. This clustering has been interpreted as evidence that long-range interactions between transcription factors or between transcription factors and nucleosomes are required for proper gene regulation [10],[11].

**Figure 4:**
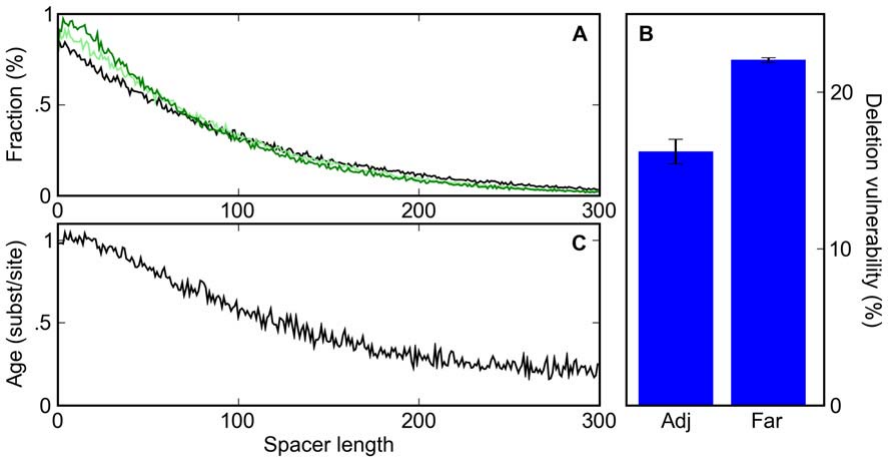
A deletion bias leads to clustering of sites and the apparent conservation of clustered sites. (A) The distribution of spacer lengths between binding sites during simulations in which 0% (black), 20% (light green), and 40% (dark green) of mutation events are indels with a 3∶2 deletion∶insertion bias. (B) The percent probability that a deletion event affecting a given binding site is accepted by our selective process for adjacent sites (Adj; sites that are touching) or far sites (Far; those with a spacer of at least twenty bases to the nearest neighboring site). (C) The distribution of the average age of binding sites as a function of their distance to their nearest neighbor shows that clustered sites appear more conserved than isolated sites, even though no such selection was applied in the simulations.

However, in our simulations, we also observed an increase in the proportion of small spacers (Figure 4A). This induced binding site clustering occurred whenever the mutation model included a bias for deletions over insertions, a known property of *Drosophila* species [24]. When simulations were run with only point mutations, or with balanced insertions and deletions, no increase in short spacers was observed.

Unlike point mutations, deletions can disrupt multiple non-overlapping binding sites. In our simulations, deletions affecting two or more sites were less than half as likely to be accepted as were deletions affecting single sites (10.5% compared to 23.2% of the time). Thus it is possible that the induced binding site clustering arises from the protective effect proximal sites have against each other's deletion (Figure 4B). Indeed, in simulations that exclusively involved deletions, tightly spaced but non-overlapping sites showed a substantial increase in half-life (Figure S3). However, in simulations with a realistic balance of mutations and indels this effect was minimal (Figure S4), as the frequency of multi-site deletions was low relative to single site deletions and point mutations.

Instead, the induced binding site clustering appears to be driven simply by the deletion of spacer DNA between sites. Since, in our simulations, deletions between sites occur more frequently than sites are lost, sites get closer together over time, distorting the distribution of inter-site distances. A corollary of this phenomenon is that sites that are observed to be close together tend to be older, and therefore more likely to be labeled as conserved, than isolated sites (Figure 4C). Thus, both binding site clustering and an apparent preferential conservation of clustered sites are expected to occur even in the absence of any selection on enhancer organization.

### A deletion bias distorts evolutionary inference

**Figure 5:**
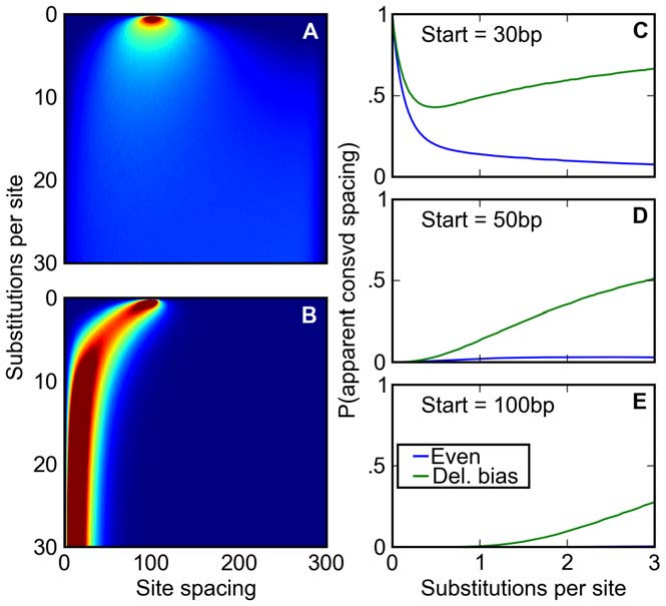
A deletion bias creates the appearance of conserved site spacing. (A,B) Following an initial starting condition where two binding sites are 100 base pairs apart, the evolution of their spacing is simulated where either (A) there is no bias towards deletions or (B) the distribution of indels approximates that found in *Drosophila*. The probability of observing the sites separated by a given distance after a given number of substitutions is shown on a scale of deep blue (zero) to deep red (≥2%). Without a deletion bias, site spacing rapidly becomes unpredictable. However, the deletion bias, on average, ratchets sites together over time, correlating any two pairs of sites' evolution. (C–E) After starting 30 (C), 50 (D), or 100 (E) base pairs apart at a speciation event, orthologous pairs of sites are subjected to a simple test of spacing conservation. If both pairs of sites are separated by a distance of 30 base pairs or less after diverging by a certain number of substitutions, their close spacing is considered ‘conserved.’ We plot the chance that, given that none of the sites themselves have degraded, this apparent conservation could be created by a neutral model. This neutral model may have a balance of insertions and deletions (blue) or a deletion bias approximating *Drosophila's* (green). When no deletion bias is present, the chance that apparently conserved spacing is explained by neutral forces decreases over time, allowing better discrimination of ‘true’ conservation via negative selection. *Drosophila*'s neutral mutation pattern not only reverses this trend (C), but also induces a substantial fraction of originally distantly spaced sites to appear to have a conserved close spacing (D, E).

Sequence features present in multiple related species are generally considered to reflect those found in the shared ancestor, whether through selection or common descent. However, the deletion bias-induced tendency for sites to get closer together over time distorts this relationship. To illustrate this, we placed two sites at a fixed distance and monitored the distance between them over time in a large number of independent simulations. With indels, but no bias towards deletions either in frequency or in average length, the intersite spacing quickly diverges between simulations (Figure 5A). However, with the observed *Drosophila* deletion bias, the spacing between sites in the different simulations is strongly correlated (Figure 5B). Thus, with a deletion bias, the spacing between sites after speciation will appear conserved and yet reflect neither selection nor the ancestral state.

To examine how this relationship between inter-site spacing and age might affect evolutionary inference, we simulated the divergence after speciation of regulatory sequences containing pairs of binding sites separated by varying distances. We then compared, at different times after divergence, the inter-site spacing in orthologous evolved sequences. Roughly following practice in the field, we considered the spacing to be “conserved” if the sites were within 30 bp in both species. Even where the starting spacing was 30 bp, the probability that it remained within 30 bp in both species in the absence of a deletion bias is small at evolutionary distances beyond one substitution per site (Figure 5C). But with a deletion bias, the probability is substantially higher, and is appreciable even for starting spacings of 50 or 100 bp (Figure 5C–5E). Thus, comparison of binding site spacing in multiple species with deletion biases will often lead to the incorrect inference that selection has acted to preserve close spacing of binding sites.

### A plausible evolutionary scenario explains positional information in a *Drosophila* enhancer

To assess whether the above-described effects could replicate the degree of binding site overlap and clustering that is observed in extant enhancers, we simulated the evolution of the well-characterized *eve* stripe 2 enhancer [23], with compositional constraints derived from the extensive biochemical and genetic literature on this enhancer. In particular we required five Krüppel, ten Bicoid, three Hunchback, five Giant [25], and a single Zelda [26] binding site (see Table 1). We also required that a certain number of sites for each factor be predicted high-affinity sites (based on the number of high-affinity sites in the *D. melanogaster* enhancer).

**Table 1:** Modeled constraint on *eve* stripe 2 enhancer.

Factor	Threshold	Count
Zelda (CAGGTAG)	9.0	1
Giant	5.0	1
Giant	2.5	4
Bicoid	7.0	3
Bicoid	4.5	7
Krüppel	8.0	1
Krüppel	7.0	2
Krüppel	4.0	2
Hunchback	7.0	1
Hunchback	5.0	2

**Figure 6:**
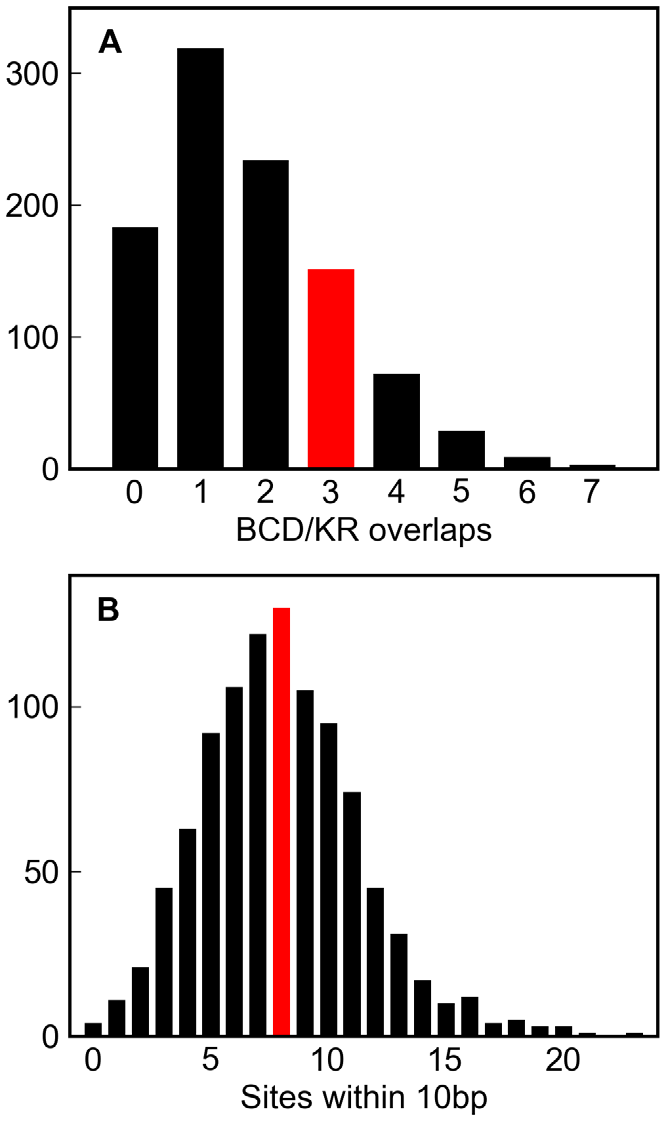
Simulations of a well-characterized *D. melanogaster* enhancer. One thousand simulations of the *eve* stripe 2 enhancer (see Methods) resulted in variable numbers of overlapping Bicoid and Krüppel sites (A, grey histogram) and sites within 10 basepairs of each other (B, grey histogram). The number of overlapping Bicoid/Krüppel site pairs, and closely spaced sites in the real *eve* stripe 2 enhancer are shown in red. That the real numbers are comfortably within the range produced by these simulations demonstrates that the higher-order structure in real *D. melanogaster* enhancers could plausibly have arisen solely from deletion biased mutation and selection to maintain binding site composition.

We simulated 1,000 replicates of this enhancer to twenty substitutions per site, and found that both the number of overlapping BCD and KR sites, and the number of sites in close proximity to others, in the real enhancer were well within the range typically generated by this architecture-free evolutionary model (Figure 6A and 6B).

## Discussion

New molecular methods and ever more sophisticated computational approaches have made significant progress towards understanding the mechanisms of gene regulation. Sequence affinities and binding sites for many transcription factors in many organisms are known, and increasing attention is now being paid to the ‘grammar’ that may link them together [27]–[29].

A common strategy in our work and that of many of our colleagues has been to infer functional constraints on enhancer activity from the apparent conservation of aspects of the organization of transcription factor binding sites within enhancers. However, the results of the simulations presented here show that many of our conclusions were based on naïve assumptions about the expected distribution of binding sites in enhancers evolving with no constraints on their organization.

### The value of simulations

In retrospect, the properties we observed are straightforward consequences of coupling selection on binding site composition with a deletion-biased mutational process. One does not need simulations to see why overlapping sites will clearly turn over less frequently than isolated sites, that a deletion bias will drive sites closer together over time, and how both phenomena distort comparative analyses.

But as self-evident as these results may appear, they have never been noted before, despite more than a decade of intense comparative genomic analysis of enhancer structure and function in *Drosophila*. Indeed, prior to performing these simulations we did not consider that the clustering of binding sites in *Drosophila* enhancers might arise from a deletion bias. We simply attempted to have our simulations accurately reflect the underlying mutational process in our simulations, with the consequences evident only in the results. This highlights the value of simulations of simple evolutionary processes in uncovering unappreciated consequences of our models and assumptions.

Furthermore, although the general effects of selection on binding site composition and of a deletion bias can be intuited, specific quantitative aspects of the model are difficult to work out analytically. For example, while we have developed a mathematical model for the effect on half-life of overlapping sites in enhancers (see Text S2), it is difficult to extend this model to enhancers with multiple sites. Simulations can answer these questions simply and effectively.

### Generality

The simulations we performed here used non-coding DNA, transcription factor binding sites, and mutation patterns from *D. melanogaster*. Interspecies differences in the composition of non-coding DNA, specificity of transcription factors, and base substitution patterns will have minimal effect on our conclusions. However, differences in the indel rate and the balance of insertions and deletions could significantly alter the existence or magnitude of the induced binding site clustering. Although the deletion biased mutation process we used in our model is often thought of as a *Drosophila*-specific phenomena, there is increasing evidence that short indels are deletion biased in all species [30]–[35]. Thus, we expect this effect to be general, although the magnitude will differ depending on the indel rate and bias (see Text S3).

### Conclusions

Lynch has eloquently argued that biologists are often too quick to assume that organismal and genomic complexity must arise from selection for complex structures and too slow to adopt non-adaptive hypotheses [12]. Our results lend additional support to this view, and extend it to show that indirect and non-adaptive forces can not only produce structure, but also create an illusion that this structure is being conserved.

We do not doubt that many aspects of transcriptional regulation constrain the location of transcription factor binding sites within enhancers. Indeed a large body of experimental evidence supports this notion, and we remain committed to identifying and characterizing these constraints. But if this process is to be fueled by comparative sequence analysis, as we believe it must be, it is essential that we give careful consideration to the neutral and indirect forces that we now know can produce evolutionary mirages of structure and function.

## Methods

### Simulation of enhancer evolution

Starting sequences 1,000 basepairs in length were generated randomly to match the base composition of *D. melanogaster* non-coding DNA, and binding sites were added to bring the starting density of sites to the specified thresholds. Mutations were sampled randomly from point mutations, insertions, and deletions. 80% of mutations were point mutations generated from an HKY85 [36] model with GC content 40% and kappa two; 12% were deletions and 8% insertions with size distributions drawn from [13]. The deletion bias (60%), and proportion of all mutations that were indels (20%), were also according to [13]. Except where noted, simulations took place for 100,000 mutation/selection rounds. To compensate for the change in the size of the enhancer when insertions and deletions occurred, bases were removed or added from the nearest edge of the sequence. New base pairs added with a 40% GC content. The simulation software was written in Python and utilizes the Motility [37] binding site identification package.

Simulations using BCD and KR used matrices from *in vitro* footprinting [38], one-hybrid assays [39], and SELEX [40], with cutoff scores chosen to match expected numbers of their sites in the *even-skipped* stripe two enhancer: 5.5, 4.9, and 4.1 for BCD and 5.6, 4.1, and 0.0 for KR for the three sources of matrices. Unless noted otherwise, simulations used matrices from the footprinting data set.

In the simulations in presented in Figure 5, we sought only to examine the evolution of site spacing over time and not the conservation and/or turnover of individual binding sites. Thus, we preconditioned in each case that neither could binding sites be generated from random sequence nor could existing binding sites be disrupted. To this end, in these simulations, all mutations affecting positions contained within existing binding sites were considered precluded by selection and discarded, and, similarly, the sequence was not scored for new binding sites created by mutations. We generated Figure 5A and 5B by simulating 980,000 300 base pair sequences to 30 substitutions per site, and Figure 5C–5E by simulating 480,000 300 base pair sequences to ten substitutions per site. In the even indels case, the distribution of insertion lengths was set equal to the distribution of deletion lengths.

Properties of the simulations were computed following a lengthy (∼30 subs/site) burn-in period that allowed the randomly generated starting model to reach equilibrium. We tested several sets of neutral mutation and selective parameters to make sure this burn-in period was sufficient (Figure S5, Figure S6, and Figure S7).

### Generation of randomized binding sites

We chose binding site lengths randomly between five and twelve. At each position, we chose a consensus nucleotide and assigned its frequency by sampling a Gaussian with mean 0.8 and standard deviation 0.2. Subsequent nucleotide frequencies were chosen similarly, each being given a frequency chosen from a Gaussian with a mean and standard deviation of 80% and 20% of the remaining probability mass, respectively. Weight matrices were constructed against a 40% GC bias and threshold scores were sampled from a uniform distribution spanning zero to the maximum scores of the sites. Information content was calculated by weighting all N-mers above the score threshold with the GC bias and subtracting the information in an N-mer of random sequence of equal length and GC bias.

### Conditional probability of overlapped sites

To find the expected probability KR and BCD sites would overlap in random DNA, we sampled random ten-mers from a 40% GC background distribution. If this sequence contained a KR site, then we added flanking sequence of length N-1, where N is the length of a BCD site. If this sequence also contained a BCD site, then we considered it as an overlap. The probability of a BCD site generating a KR site was found in an analogous manner. The post-selection conditional probability was directly calculated by simulating an enhancer with five sites for each transcription factor as described above and counting observations of singleton and overlapped binding sites.

### Half-lives of binding sites

We determined the half-lives of sets of binding sites by randomly sampling individual sites in our simulations and observing their degradation as the simulations progressed. Our data consisted of simulations of 1,000 enhancers, each run for 30,000 iterations. For each enhancer, after a burn-in period of 10,000 iterations, we took a ‘snapshot’ of the binding sites present every 3,000 iterations. In each subsequent iteration of the simulation, the presence or absence of each binding site in the snapshot was assessed: if it had been destroyed by a point mutation or indel in that iteration, then a site ‘death’ was recorded. This process was repeated for 2,000 post-snapshot iterations of the simulation.

### Generation of the even-skipped stripe two enhancer

We used one-hybrid binding sequences for Hunchback, Giant, Bicoid, and Krüppel from [39] and created weighted matrices as described. We used the same methods to generate a Zelda-consensus matrix from the sequences listed in [41]. Our enhancer sequence and matrices are available in Dataset S1. In order to determine the required number of sites for each matrix, we assessed the number of hits it had to the *eve* stripe 2 sequence at several score cutoffs. If the number of hits at a given score cutoff exceeded the number expected by chance, then this number/score cutoff pair was accepted as a requirement, provided that it did not substantially increase the total required number of sites for that factor beyond that described in [23]. The constraint on the enhancer is described in Table 1.

## Supporting Information

Figure S1 The half-life of overlapping or singleton sites computed using BCD and KR specificity matrixes from [39].
(4.57 MB TIF)

Figure S2 The half-life of overlapping or singleton sites computed using BCD and KR specificity matrixes from our unpublished SELEX data.
(4.57 MB TIF)

Figure S3 In simulations that exclusively involved deletions, tightly-spaced but non-overlapping sites (solid lines) showed a substantial increase in half-life over isolated sites (dotted lines).
(4.57 MB TIF)

Figure S4 In simulations using the actual *D. melanogaster* substitution and indel patterns, the protective effect of deletions is minimal, as the frequency of multi-site deletions was low relative to single site deletions and point mutations.
(4.57 MB TIF)

Figure S5 The probability of a KR site containing a BCD site (blue) or vice-versa (red), as described in Figure 1, is plotted as a function of time for rapid (top), normal (middle), and slow (bottom) turnover rates. Rapid turnover was induced by lowering the necessary score thresholds for BCD and KR to 4.5 and 4.6, respectively, and slow turnover induced by raising the necessary score thresholds to 6.5 and 6.6. These simulations have no insertions and deletions.
(4.68 MB TIF)

Figure S6 The probability of a KR site containing a BCD site (blue) or vice-versa (red), as described in Figure 1, is plotted as a function of time for rapid (top), normal (middle), and slow (bottom) turnover rates. Rapid turnover was induced by lowering the necessary score thresholds for BCD and KR to 4.5 and 4.6, respectively, and slow turnover induced by raising the necessary score thresholds to 6.5 and 6.6. In these simulations 20% of mutations are indels. The proportion of indels that are deletions is 50% (left), 60% (middle), and 80% (right).
(8.04 MB TIF)

Figure S7 The probability of a KR site containing a BCD site (blue) or vice-versa (red), as described in Figure 1, is plotted as a function of time for rapid (top), normal (middle), and slow (bottom) turnover rates. Rapid turnover was induced by lowering the necessary score thresholds for BCD and KR to 4.5 and 4.6, respectively, and slow turnover induced by raising the necessary score thresholds to 6.5 and 6.6. In these simulations 40% of mutations are indels. The proportion of indels that are deletions is 50% (left), 60% (middle), and 80% (right).
(8.04 MB TIF)

Text S1 Comparison of threshold and population genetic model for enhancer evolution.
(0.63 MB PDF)

Text S2 An evolutionary model of overlapping sites predicts a reduced nucleotide substitution rate.
(0.33 MB PDF)

Text S3 The frequency and size of insertions and deletions affect site clustering.
(0.41 MB PDF)

Dataset S1 Transcription factor matrices and enhancer sequence used in this study.
(0.01 MB CDX)
