# Supplementary figures and images for: Evolutionary Mirages: Selection on Binding Site Composition Creates the Illusion of Conserved Grammars in Drosophila Enhancers
Source: PLoS Genet. 2010 Jan 22;6(1):e1000829. doi: 10.1371/journal.pgen.1000829 (PMC2809757; doi:10.1371/journal.pgen.1000829)

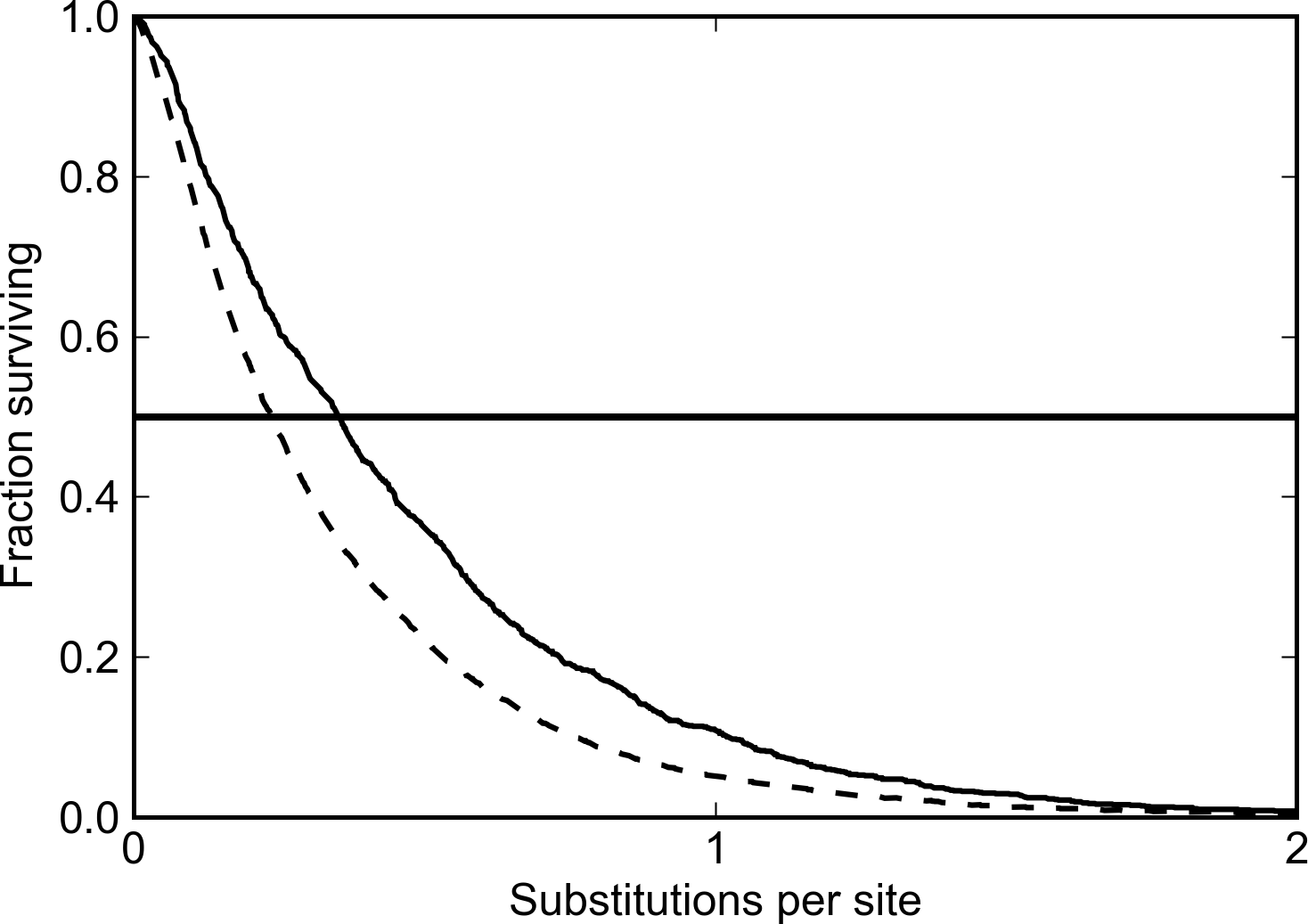

Supplement: Figure S1 — The half-life of overlapping or singleton sites computed using BCD and KR specificity matrixes from [39]. (4.57 MB TIF) [file pgen.1000829.s001.tif]

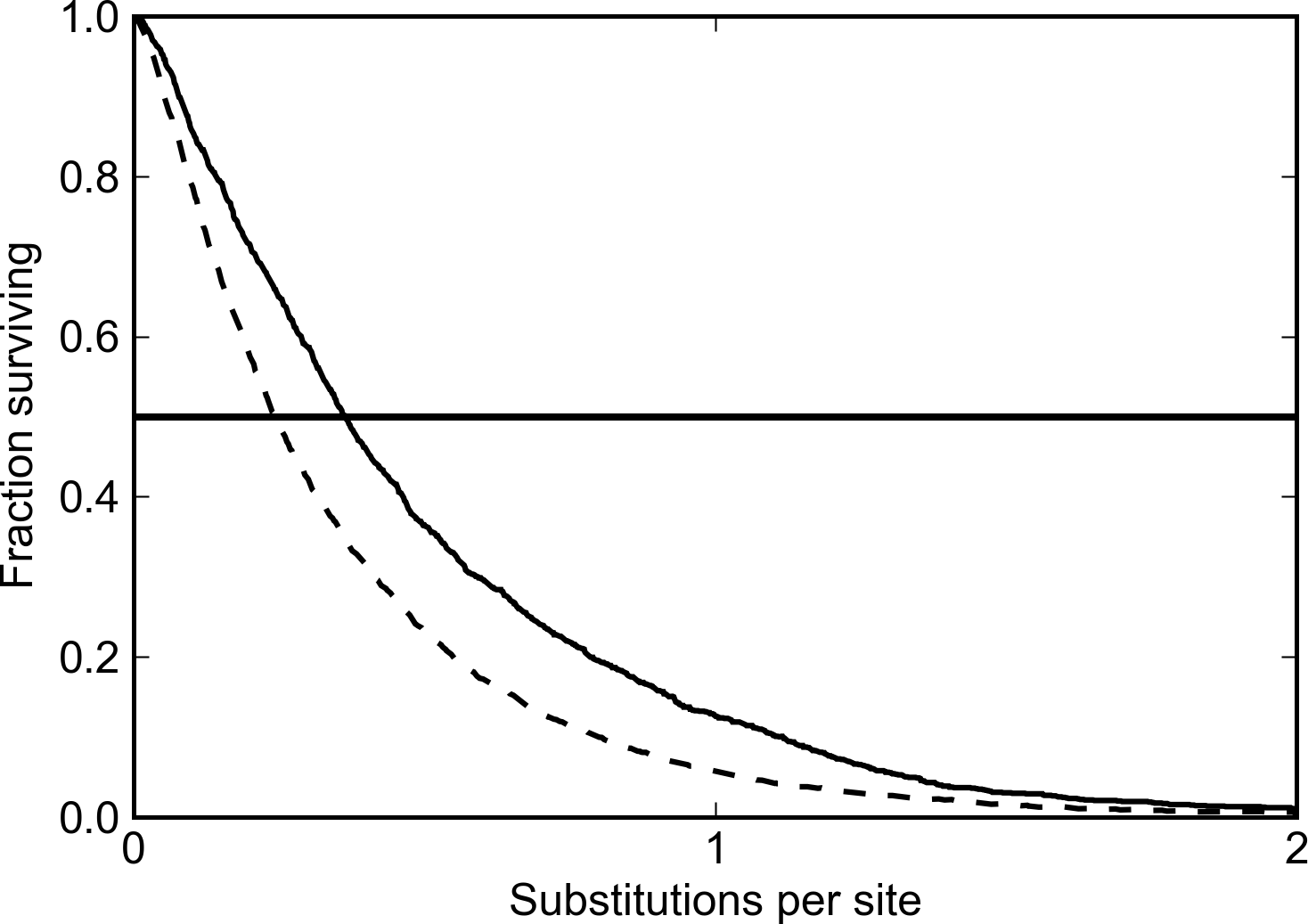

Supplement: Figure S2 — The half-life of overlapping or singleton sites computed using BCD and KR specificity matrixes from our unpublished SELEX data. (4.57 MB TIF) [file pgen.1000829.s002.tif]

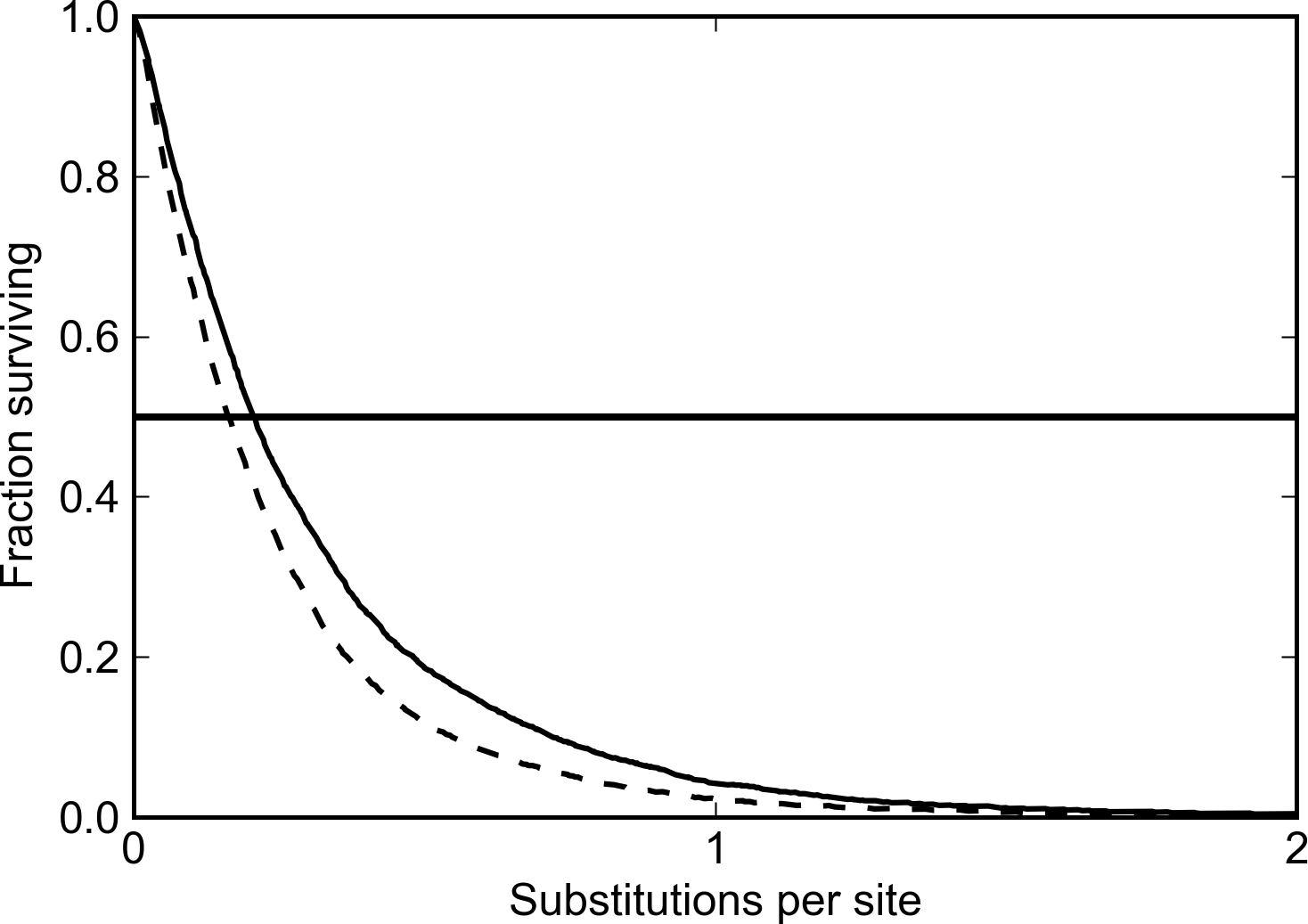

Supplement: Figure S3 — In simulations that exclusively involved deletions, tightly-spaced but non-overlapping sites (solid lines) showed a substantial increase in half-life over isolated sites (dotted lines). (4.57 MB TIF) [file pgen.1000829.s003.tif]

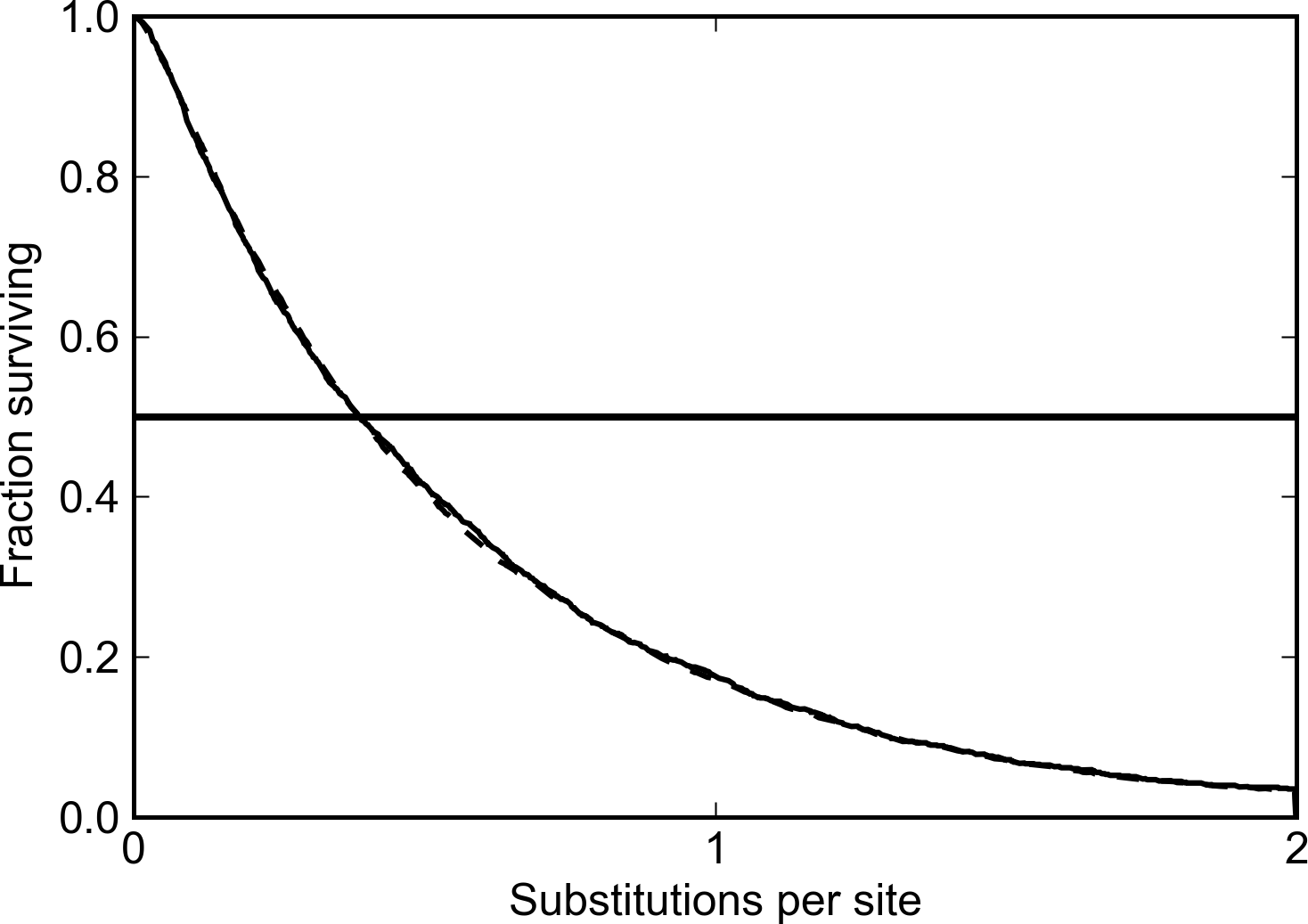

Supplement: Figure S4 — In simulations using the actual D. melanogaster substitution and indel patterns, the protective effect of deletions is minimal, as the frequency of multi-site deletions was low relative to single site deletions and point mutations. (4.57 MB TIF) [file pgen.1000829.s004.tif]

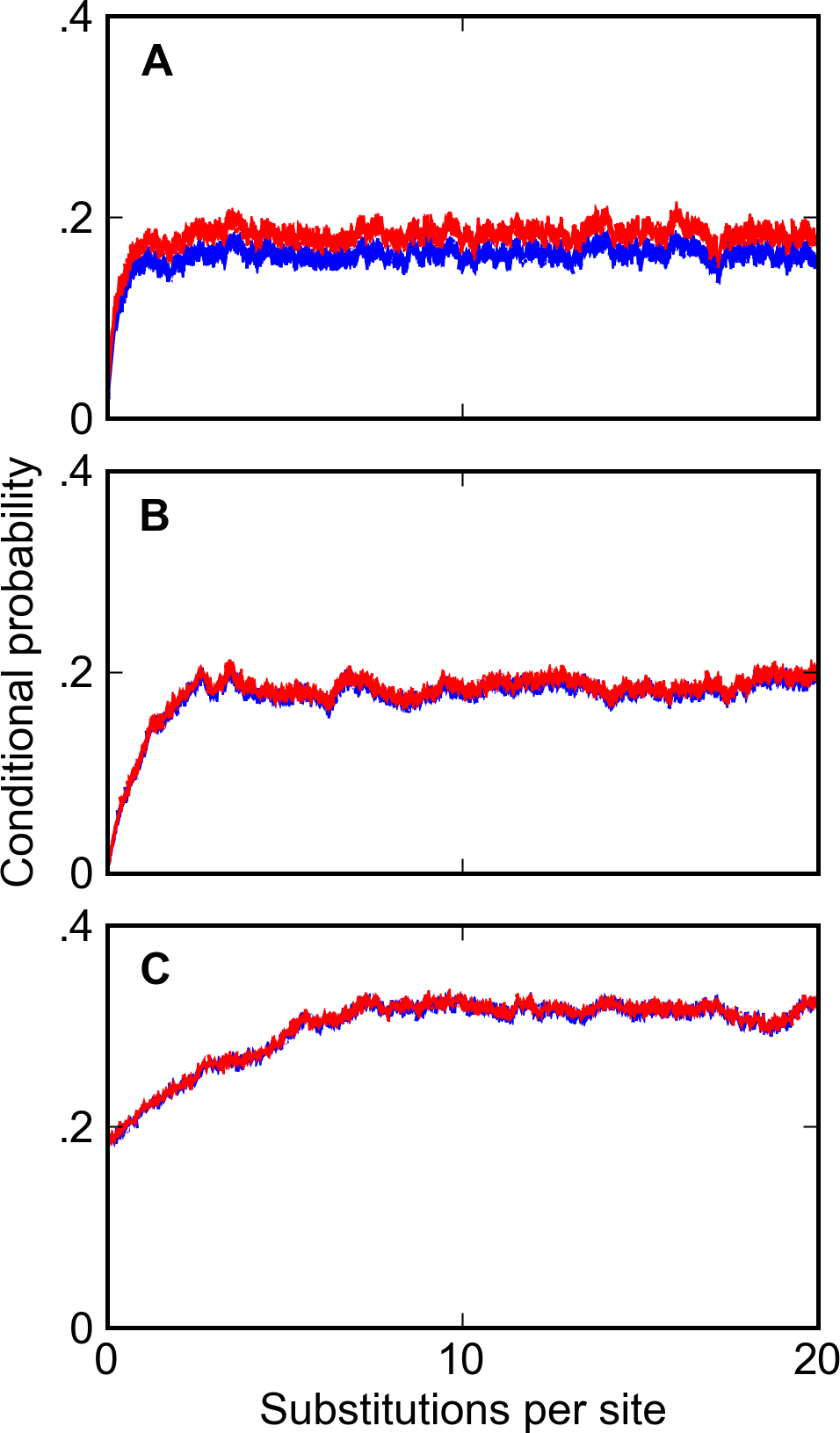

Supplement: Figure S5 — The probability of a KR site containing a BCD site (blue) or vice-versa (red), as described in Figure 1, is plotted as a function of time for rapid (top), normal (middle), and slow (bottom) turnover rates. Rapid turnover was induced by lowering the necessary score thresholds for BCD and KR to 4.5 and 4.6, respectively, and slow turnover induced by raising the necessary score thresholds to 6.5 and 6.6. These simulations have no insertions and deletions. (4.68 MB TIF) [file pgen.1000829.s005.tif]

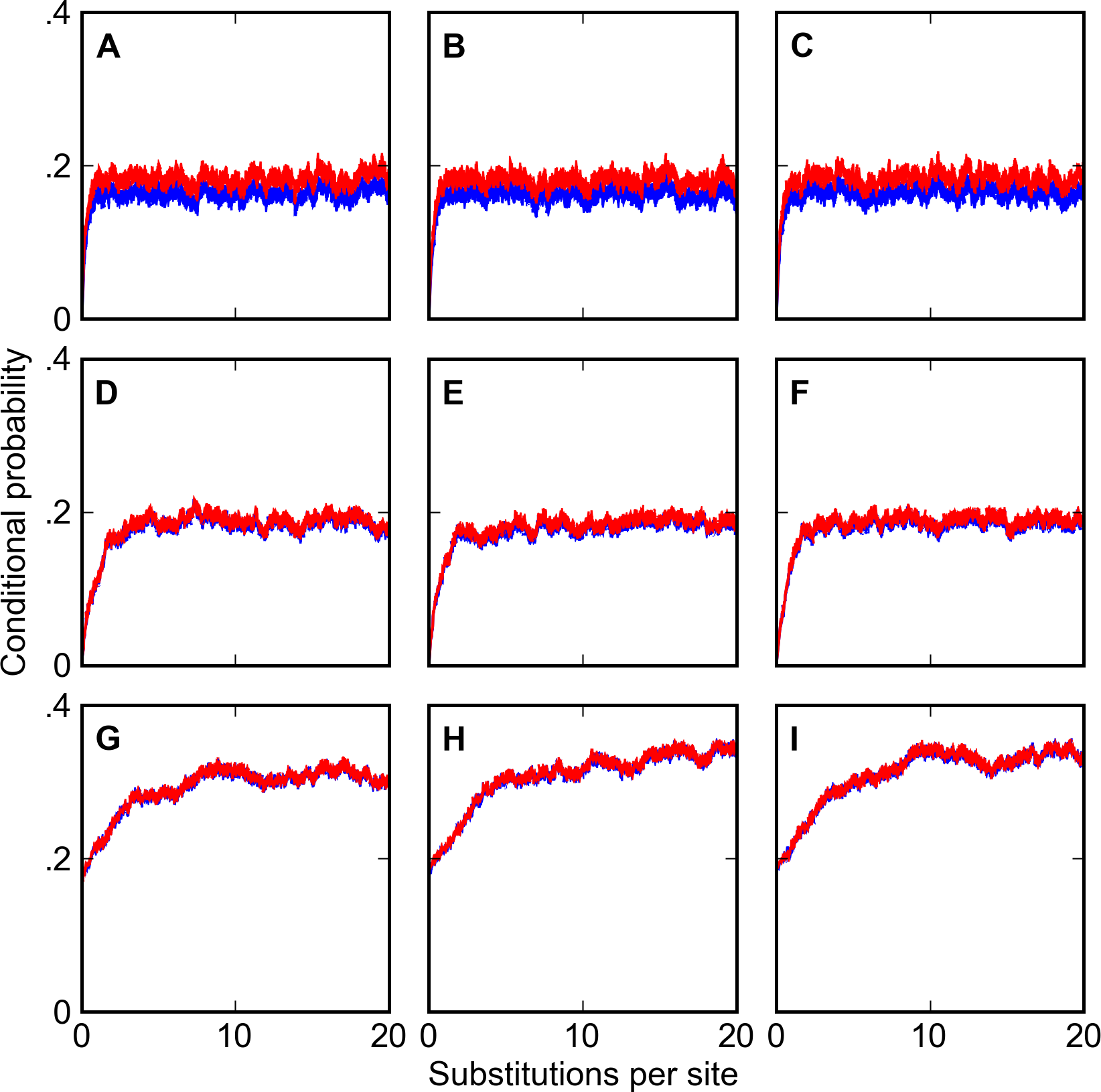

Supplement: Figure S6 — The probability of a KR site containing a BCD site (blue) or vice-versa (red), as described in Figure 1, is plotted as a function of time for rapid (top), normal (middle), and slow (bottom) turnover rates. Rapid turnover was induced by lowering the necessary score thresholds for BCD and KR to 4.5 and 4.6, respectively, and slow turnover induced by raising the necessary score thresholds to 6.5 and 6.6. In these simulations 20% of mutations are indels. The proportion of indels that are deletions is 50% (left), 60% (middle), and 80% (right). (8.04 MB TIF) [file pgen.1000829.s006.tif]

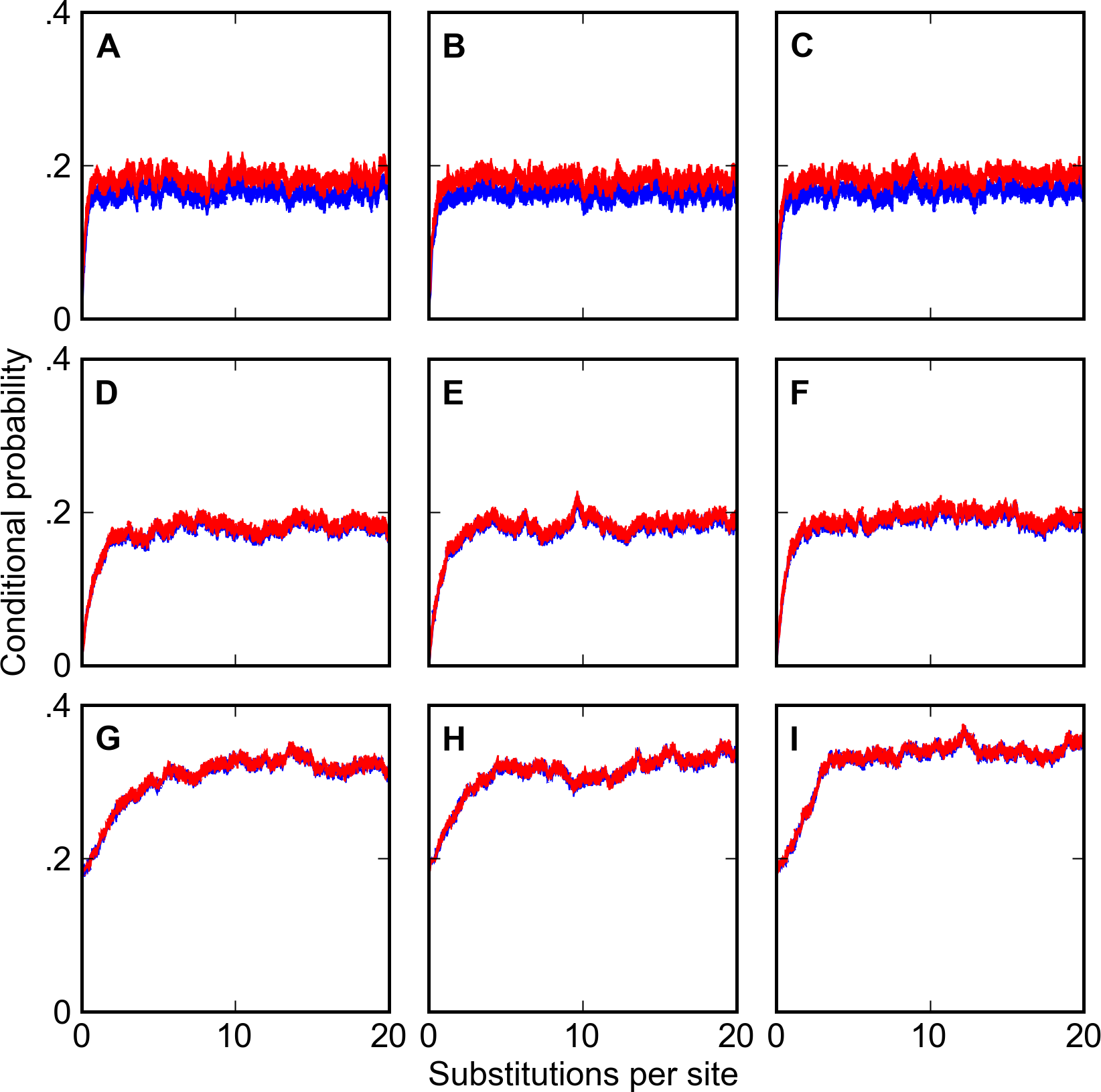

Supplement: Figure S7 — The probability of a KR site containing a BCD site (blue) or vice-versa (red), as described in Figure 1, is plotted as a function of time for rapid (top), normal (middle), and slow (bottom) turnover rates. Rapid turnover was induced by lowering the necessary score thresholds for BCD and KR to 4.5 and 4.6, respectively, and slow turnover induced by raising the necessary score thresholds to 6.5 and 6.6. In these simulations 40% of mutations are indels. The proportion of indels that are deletions is 50% (left), 60% (middle), and 80% (right). (8.04 MB TIF) [file pgen.1000829.s007.tif]
