# Supplementary material for: Evolutionary Mirages: Selection on Binding Site Composition Creates the Illusion of Conserved Grammars in Drosophila Enhancers
Source: PLoS Genet. 2010 Jan 22;6(1):e1000829. doi: 10.1371/journal.pgen.1000829 (PMC2809757; doi:10.1371/journal.pgen.1000829)
Supplement: Text S1 — Comparison of threshold and population genetic model for enhancer evolution. (0.63 MB PDF) [file pgen.1000829.s008.pdf]

Text S1. Comparison of threshold and population genetic model for enhancer evolution

Appendix to Lusk and Eisen, "Evolutionary mirages: selection on binding site composition creates the illusion of conserved grammars in *Drosophila* enhancers".

The simulations presented in the main text consider a single sequence evolving with either a fitness of 1 if they have a sufficient number of sites or 0 if they do not. To examine whether any of our results were a product of this strict threshold, we performed a whole-population simulation in which enhancers with suboptimal binding site compositions were viable but received a fitness penalty. We did not find any appreciable differences between the results of these simulations and those presented in the main text.

In these population simulations, each enhancer was assigned a fitness of one if it had at least five Bicoid sites and five Krüppel sites. Mutations to enhancers that brought the number of sites below these requirements were given a selective penalty defined as the number of missing sites multiplied by a penalty factor s. In the selection step, alleles were resampled according to their selective penalties. This method gives deleterious alleles missing binding sites some chance of being fixed in the population.

We ran our simulations of a population of 10,000 enhancers for five values of the selective penalty factor s. As expected, at $s = 0$, representing no penalty for suboptimal site densities, the average number of sites in enhancers rapidly approaches the background frequency (Figure 1). As the penalty increases, so does the site density, and for values of $s = 0.01$ and 0.001 the average density is close to the threshold. Since comparative genomic analyses show that the density of sites in enhancers is conserved over long evolutionary distances, these high values of s

are the most realistic. At these values, the rate of turnover, spatial distribution of sites, and enrichment of overlapping binding sites were indistinguishable from those observed in the simpler threshold-based model (Figure 2, 3 and 4), allowing us to take advantage of the greater computational tractability of the threshold model in the main text.

Although not relevant for the main text, the intermediate value of s = .0001 provided an interesting case. Compared to simulations run under more stringent selective penalties, not only was the rate of loss per site greater, but also, less intuitively, the rate of site gain from neutral sequence was greater (Figure 3). As the rate of generation of alleles containing new sites must remain roughly the same between these simulations, we reasoned that the probability of fixation of alleles containing new sites must be higher. Indeed, when a more stringent selective penalty was used, virtually no new alleles arose with a selective advantage over the major allele, as all alleles at appreciable frequency already have a sufficient number of sites (data not shown). At this lesser penalty, where the major allele typically has an insufficient number of sites, alleles containing new sites can carry a selective advantage. While this phenomenon increased the rate of turnover, it had no effect on the increased enrichment of overlapping binding sites.

Methods

Each simulation was started from a population containing 10,000 identical 1,000 base pair enhancers with five Bicoid sites and five Krüppel sites meeting the score cutoffs described in the main text. For each generation's mutation step, we used the mutation rate described in [1] to arrive at a mutation rate of 7.56*10^-6 mutations per enhancer per generation. Assuming that no single enhancer would mutate twice in a single generation, we sampled from a poisson to determine the number of new alleles to create. Each new allele was generated from a randomly

selected enhancer by sampling from the mutation distributions described in the main text, with a 20% chance of creating an indel and a 60% chance of creating a deletion if an indel was chosen.

In each generation's selection step, we sampled 10,000 new alleles from a multinomial distribution. The parameters of this distribution were determined by creating a weight for each allele defined as that allele's current-generation count multiplied by its selective penalty, these weights then being normalized to sum to one.

For each tested penalty factor, seventy-five replicates were tested to 1,322,750,000 generations, the time necessary for 10,000 neutral mutations to reach fixation. Every 5,291,000 generations, or forty fixed neutral mutations, the major allele was recorded, each time being surveyed for binding sites for Bicoid and Krüppel. This chain of alleles was aligned using FSA [2], allowing us to determine the amount of lost and gained sites per substitution. Although we expect alignment errors at this distance to be minimal, to best compare these results with the model of the main text, we output the sequence of those simulations every 40 mutation-selection iterations and created a similar chain of aligned sequences from which gain and loss statistics were derived.

To determine the count and spacing distribution of sites and the enrichment of overlapped sites, major alleles were sampled at 2.5, 5.0, 7.5, and 10.0 substitutions per site. 95% confidence intervals for number of sites, turnover rate, site spacing, and overlap effect were calculated by resampling the data over 1,000 bootstrap replicates. Comparisons between spacer length distributions were made between the cutoff model and the population genetic model using Pearson's chi-squared test.

References

1. Haag-Liautard C, Dorris M, Maside X, Macaskill S, Halligan DL et al (2007). Direct estimation of per nucleotide and genomic deleterious mutation rates in Drosophila. *Nature* 445: 82-85.

2. Bradley RK, Roberts A, Smoot M, Juvekar S, Do J et al (2009). Fast statistical alignment. *PLoS Comput Biol* 5: e1000392.

Figures

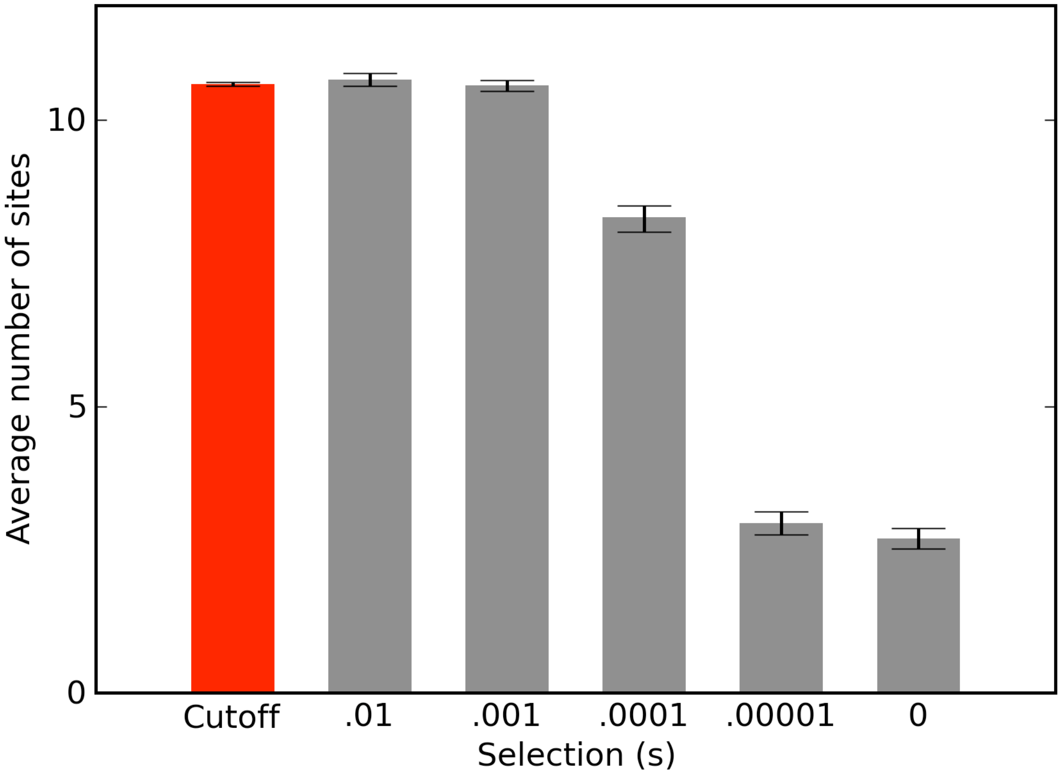

Figure 1. The average number of sites depends on the selective penalty for missing sites.

s greater than or equal to .001 is necessary to maintain the required number of sites (10). At these values, the average number of sites is not distinguishable from the average number given by the cutoff model.

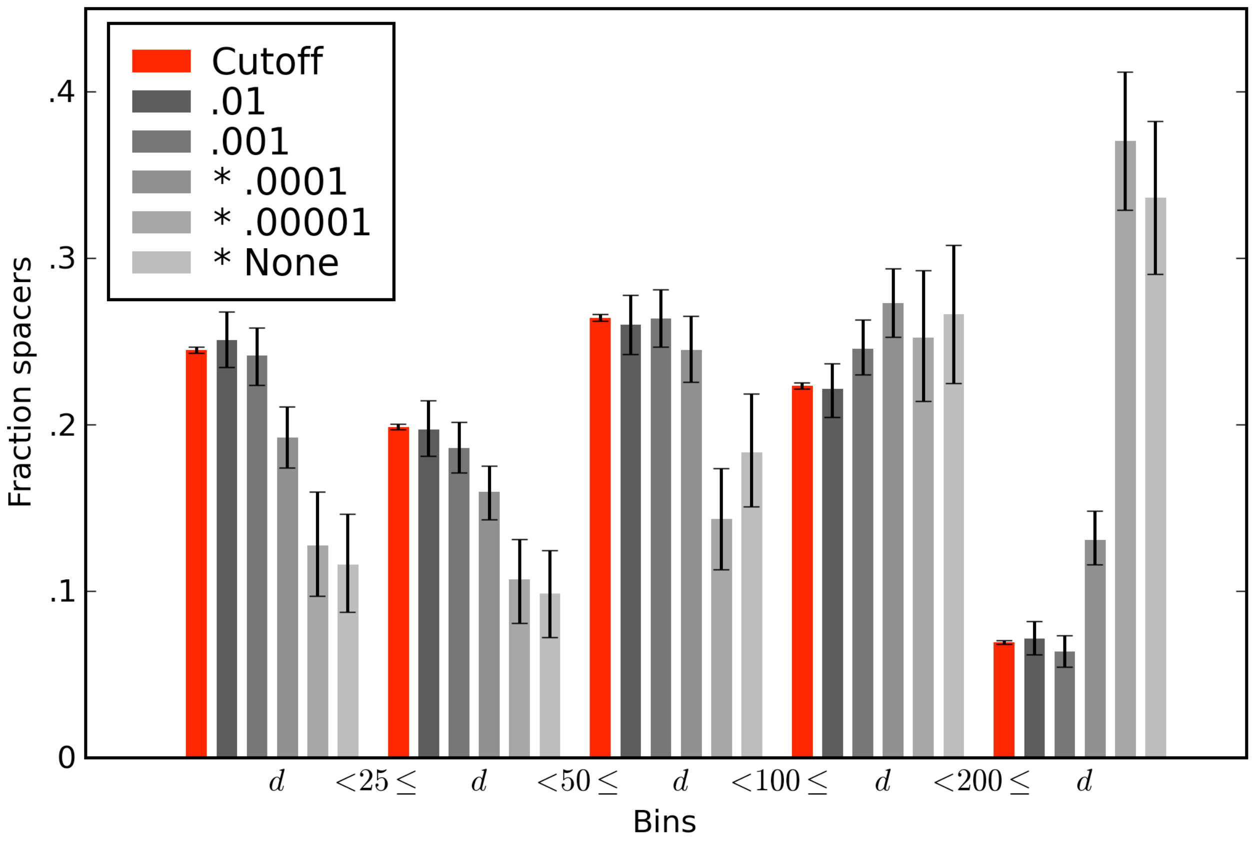

Figure 2. A population-genetic model does not affect the spatial distribution of binding sites.

Spacer elements between binding sites were divided into five distance bins, and the fraction of all spacers in each bin was plotted for the cutoff model and five population genetic models with different values of s (legend). An * denotes a significant difference from the cutoff model at α = .05.

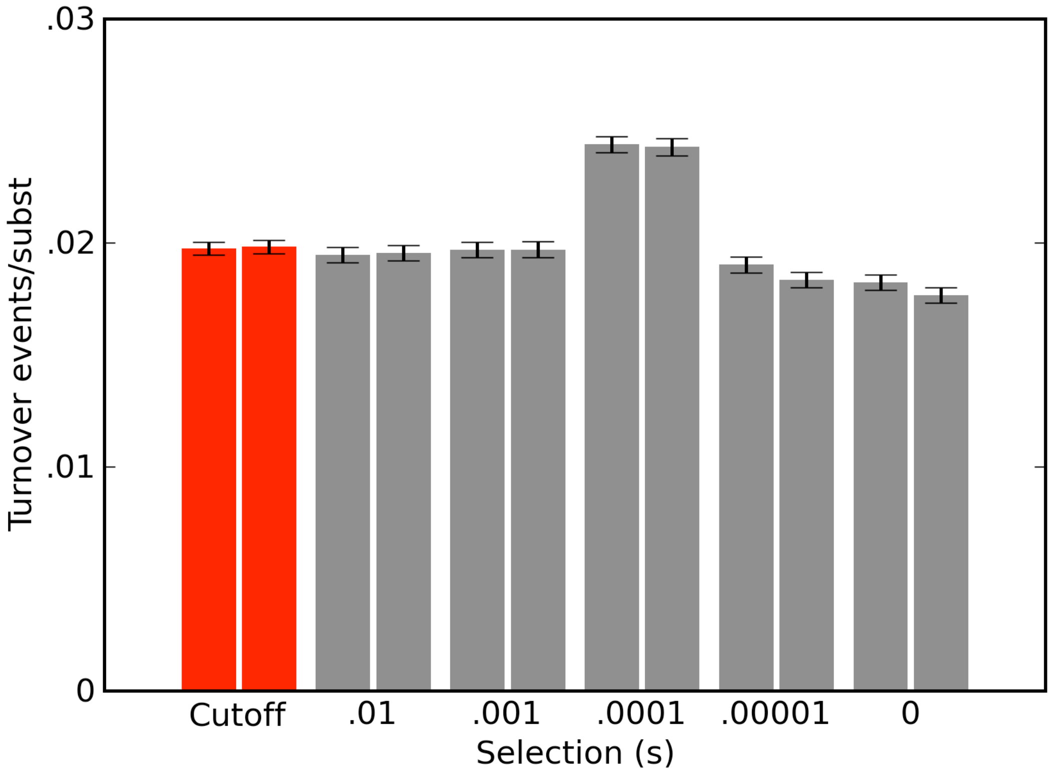

Figure 3. Rates of turnover are dependent upon selective penalty.

For each value of *s*, rates of binding site loss (left) and gain (right) per neutral substitution were plotted. At values of *s* sufficient to maintain binding site composition, the rate of turnover is not significantly different from that observed under the cutoff model.

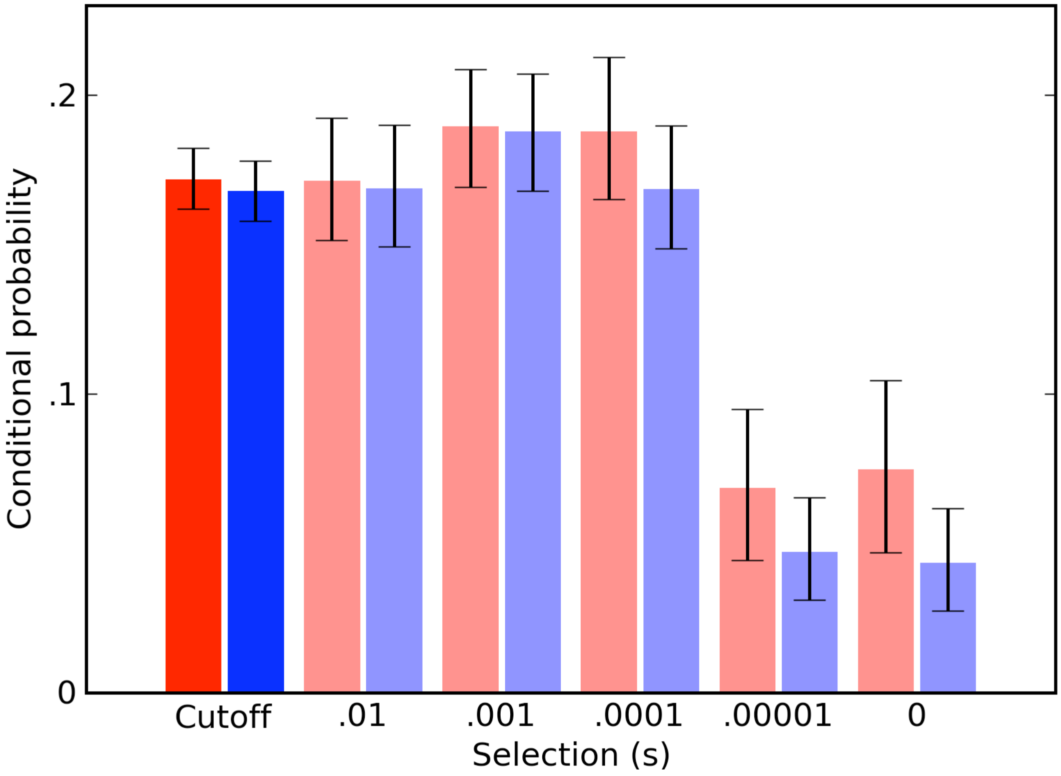

Figure 4. Overlapping binding sites are enriched in a population genetic model.

The post-simulation probability of observing a Krüppel site conditioned on seeing a Bicoid site (blue) and a Bicoid site conditioned on seeing a Krüppel site (red) is similar to that observed in the cutoff model when the selective penalty is sufficient to markedly increase the number of binding sites (s greater than or equal to .0001).
