# Supplementary material for: Evolutionary Mirages: Selection on Binding Site Composition Creates the Illusion of Conserved Grammars in Drosophila Enhancers
Source: PLoS Genet. 2010 Jan 22;6(1):e1000829. doi: 10.1371/journal.pgen.1000829 (PMC2809757; doi:10.1371/journal.pgen.1000829)
Supplement: Text S2 — An evolutionary model of overlapping sites predicts a reduced nucleotide substitution rate. (0.33 MB PDF) [file pgen.1000829.s009.pdf]

Text S2. An evolutionary model of overlapping sites predicts a reduced nucleotide substitution rate

Appendix to Lusk and Eisen, “Evolutionary mirages: selection on binding site composition creates the illusion of conserved grammars in *Drosophila* enhancers”.

As an alternative test of overlapping versus singleton site conservation, we extended a base-specific model of binding site evolution to the case of overlapping binding sites. To this end, we made the following two assumptions: first, we assumed that if two binding sites overlap but do not have a biologically meaningful interaction, then mutations in the overlapped region should behave as simultaneous mutations in two non-overlapping binding sites. Second, we assumed that selection pressure is additive. We used the framework developed by Halpern and Bruno [1], which has been shown to accurately model the evolution of individual transcription factor binding sites [2-5], to calculate the selection coefficients of every possible mutation as made to each individual site, and then we combined these coefficients to arrive at the coefficient for every possible mutation in the overlapped site. We then used these coefficients then determine position-specific rates of substitution according to classic relationships developed by Kimura [6].

We used the above described model to derive rates of substitution relative to the neutral rate for an overlapped Bicoid/Krüppel site. In positions where the two factors share a nucleotide preference, the substitution rate is strongly lowered (fig. 1), and the substitution in the overlapped region had substitution rates as low as 3.4% of the predicted single-factor rate (fig. 2). This result was consistent with longer half-lives of overlapping sites in our simulations.

Methods

According to [1], for base frequencies π and neutral substitution rates p, 2Ns for any given mutation is:

$$2Ns = \log(\frac{\pi_b p_{ba}}{\pi_a p_{ab}}) \quad (1)$$

According to the equations of Kimura [24], the fixation probability of such an allele is:

$$f_{ab} \approx \frac{2s}{1 - e^{-2Ns}} \quad (2)$$

Assuming additive selection, the rates of substitution for an overlapped region of two sites are:

$$fab \propto \frac{\log(HB1) + \log(HB2)}{1 - e^{-\log(HB1) - \log(HB2)}} \quad (3)$$

where:

$$HB = \frac{\pi_b p_{ba}}{\pi_a p_{ab}} \quad (4)$$

References

1. Halpern A, Bruno W (1998) Evolutionary distances for protein-coding sequences: modeling site-specific residue frequencies. *Mol Biol Evol* 15(7): 910-917.

2. He X, Ling X, Sinha S (2009) Alignment and prediction of cis-regulatory modules based on a probabilistic model of evolution. *PLoS Comput Biol* 5(3): e1000299.

3. Doniger S, Fay J (2007) Frequent Gain and Loss of Functional Transcription Factor Binding Sites. *PLoS Comput Biol* 3(5): e99.

4. Moses A, Pollard D, Nix D, Iyer V, Li X et al (2006) Large-scale turnover of functional transcription factor binding sites in Drosophila. *PLoS Comput Biol* 2(10): e130.

5. Moses A, Chiang D, Kellis M, Lander E, Eisen M (2003) Position specific variation in the rate of evolution in transcription factor binding sites. *BMC Evol Biol* 3: 19.

6. Kimura M (1962) On the probability of fixation of mutant genes in a population. *Genetics* 47: 713-719.

Figures

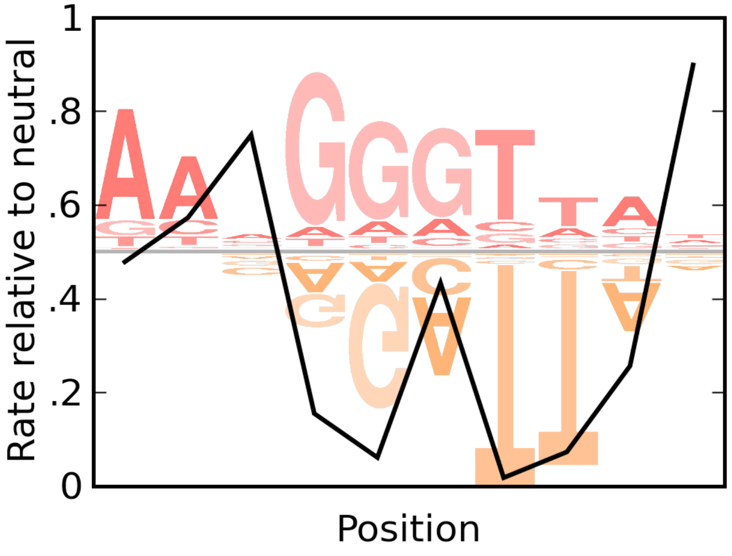

Figure 1. Aligned nucleotide preferences lower substitution rate.

Predicted rates of evolution of an overlapping Bicoid/Krüppel binding site, with the sequence logos of Krüppel (top, red) and Bicoid (bottom, orange) in the background. The rate (black) is taken relative to the expected neutral rate at that position.

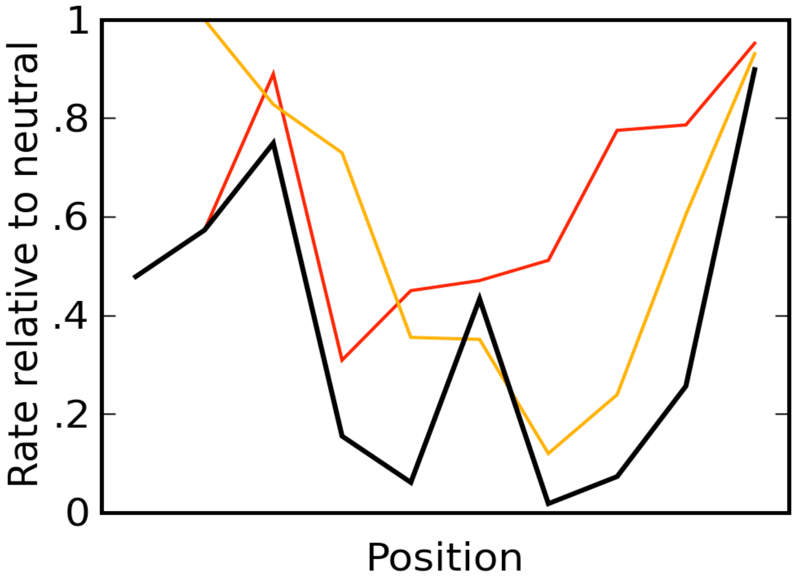

Figure 2. Site overlap strongly reduces substitution rate

Predicted rates of evolution of a Krüppel site (red), a Bicoid site (orange) and an overlapping Bicoid/Krüppel site (black).
