# Supplementary material for: Evolutionary Mirages: Selection on Binding Site Composition Creates the Illusion of Conserved Grammars in Drosophila Enhancers
Source: PLoS Genet. 2010 Jan 22;6(1):e1000829. doi: 10.1371/journal.pgen.1000829 (PMC2809757; doi:10.1371/journal.pgen.1000829)
Supplement: Text S3 — The frequency and size of insertions and deletions affect site clustering. (0.41 MB PDF) [file pgen.1000829.s010.pdf]

Text S3. The frequency and size of insertions and deletions affect site clustering

Appendix to Lusk and Eisen, "Evolutionary mirages: selection on binding site composition creates the illusion of conserved grammars in *Drosophila* enhancers".

Several recent works have proposed the importance of overlapping and locally clustered sites within *Drosophila* [1-4], and so we parameterized our neutral mutation model to approximate the patterns of point mutations, insertions, and deletions found within that clade. Although we expected that the enrichment and increased conservation of overlapping sites should largely depend on the sequence specificities of the two transcription factors involved, it was clear that the spatial arrangement of binding sites should be informed by the parameters of the neutral mutation model, in particular the indel rate and the bias, if any, of indels towards deletions. In order to generalize the quantitative conclusions of the main text beyond *Drosophila*, we trained a predictive model of binding site arrangement on simulated enhancers evolved under a wide range of these parameters. We were able to accurately predict the divergence of spatial distributions of binding sites within these enhancers from that within indel-free enhancers using only the frequencies of deletions and insertions (Figure 1).

To highlight this effect, we chose to investigate in greater detail several species whose patterns of indels have been characterized. We simulated the evolution of enhancers according to parameters derived from *C. elegans*, which has a high rate of DNA loss, mammals, and two species of grasshoppers, one of which has a particularly low rate of DNA loss [5]. In addition, we investigated the effect of another set of indel parameters for *D. melanogaster* different from that used in the main text. In these simulations, not only the frequencies of insertions and deletions but also their average size were incorporated. We found, again, that while in each case

the distribution of spacer elements was skewed, the magnitude of this skew varied widely depending on the choice of parameters: *C. elegans* and the alternate parameterization of *D. melanogaster* showed a substantially stronger enrichment of locally clustered sites than observed in the main text, while other species showed a weaker enrichment (Figure 2).

Methods

To train the model, we performed 750 simulations for each pair of indel mutation parameters shown in fig. 1. Each simulation evolved a 1,000bp enhancer containing 10 Kruppel sites (score > 5.6) for 30,000 mutation-selection rounds (simulation details are available in the main text). In these simulations, to better allow interpretation an even deletion bias, insertions and deletions were of equal average length, their lengths both being drawn from the distribution of deletion lengths described in the main text. Spacers between non-overlapping binding sites were binned according to cutoffs 25, 50, 75, 100, 150, 200, 250, 300, and 500 base pairs. The Kullback-Leibler divergence was calculated between each of these samples and a 'reference' 5,000-replicate set derived from simulations without indels. We fit a linear model relating the KL divergence to the insertion and deletion frequencies using R (ID is indel frequency, DB is deletion bias, and KL is KL divergence):

$$KL = -.147*ID + .101*DB + .454*ID*DB - .044$$

The highlighted species used indel rate, average indel sizes, and deletion bias data from [5]. The indel sizes were transformed into geometric distributions from which lengths were sampled during the simulations.

References

1. Hare EE, Peterson BK, Iyer VN, Meier R, Eisen MB (2008) Sepsid even-skipped enhancers are functionally conserved in Drosophila despite lack of sequence conservation. *PLoS Genet* 4: e1000106.

2. Kim J, He X, Sinha S (2009) Evolution of regulatory sequences in 12 Drosophila species. *PLoS Genet* 5: e1000330.

3. Makeev VJ, Lifanov AP, Nazina AG, Papatsenko DA (2003) Distance preferences in the arrangement of binding motifs and hierarchical levels in organization of transcription regulatory information. *Nucleic Acids Res* 31: 6016-26.

4. Papatsenko D, Goltsev Y, Levine M (2009) Organization of developmental enhancers in the Drosophila embryo. *Nucleic Acids Res* 37(17):5664-7.

5. Petrov DA, Sangster TA, Johnston JS, Hartl DL, Shaw KL (2000) Evidence for DNA loss as a determinant of genome size. *Science* 287: 1060-2.

6. Tanay A, Siggia ED (2008) Sequence context affects the rate of short insertions and deletions in flies and primates. *Genome Biol* 9: R37.

Figures

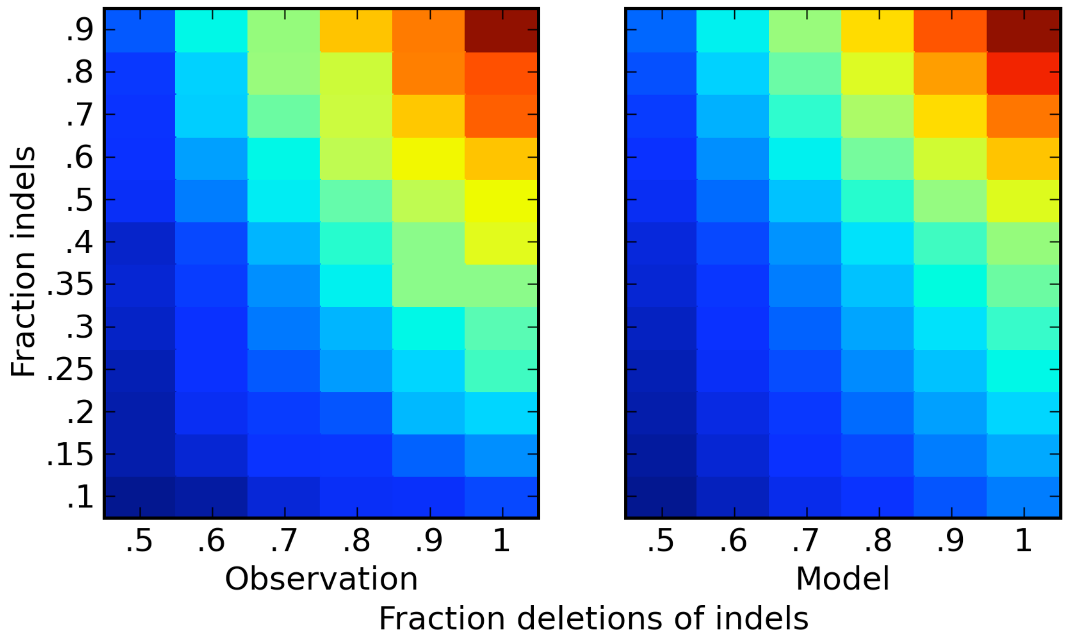

Figure 1. Deviation in spacing distribution is predictable by a linear model.

Kullback-Leibler divergence is plotted by color from a minimum of .0145 (deep blue) to a maximum of .334 (deep red).

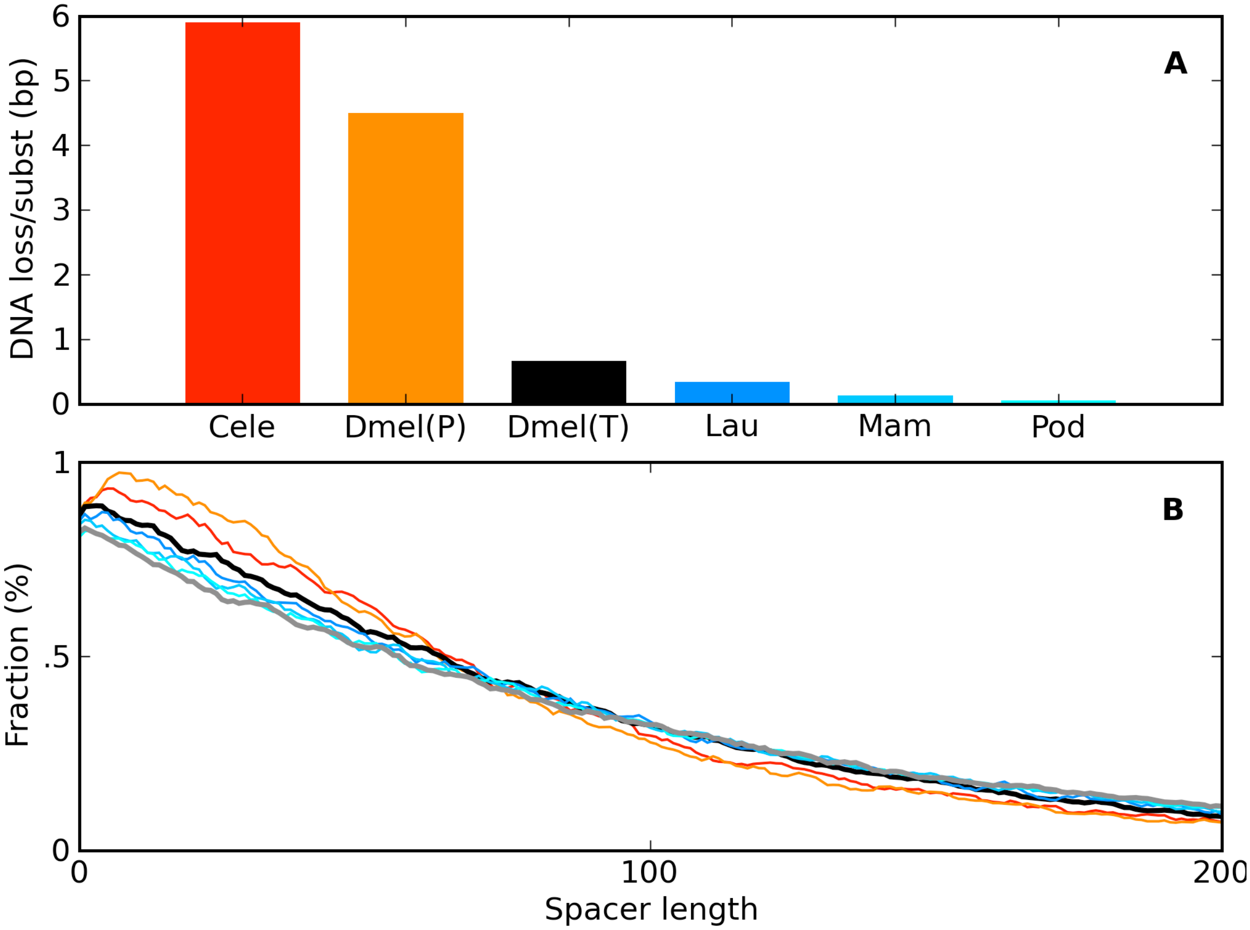

Figure 2. Spacing distribution skew is different in different organisms and depends on the rate of DNA loss.

A. DNA loss, as calculated and described in [5]. Cele is *C. elegans*, Lau and Pod refer to *Laupala* and *Podisma* grasshoppers, Mam is mammals, and Dmel(P) and Dmel(T) refer to *D. melanogaster* as described in [5] and [6], respectively. The main text used the parameters of Dmel(T). B. Spacer length distribution averaged over five base pair windows. Colors are as in (a), except for the gray line, which refers to simulations without indels.
